# Increased Frequency of Copy Number Variations Revealed by Array Comparative Genomic Hybridization in the Offspring of Male Mice Exposed to Low Dose-Rate Ionizing Radiation

**DOI:** 10.3390/ijms222212437

**Published:** 2021-11-18

**Authors:** Keiji Ogura, Yoshiko Ayabe, Chihiro Harada, Ignacia Braga Tanaka, Satoshi Tanaka, Jun-ichiro Komura

**Affiliations:** 1 Department of Radiobiology, Institute for Environmental Sciences, Aomori 039-3213, Japan; ayabe445@affrc.go.jp (Y.A.); tanakaib@ies.or.jp (I.B.T.III); tanakas@ies.or.jp (S.T.); junkom@ies.or.jp (J.-i.K.); 2 JAC Co., Ltd., Tokyo 101-0051, Japan; c.harada@jac-co.co.jp

**Keywords:** ionizing radiation, low dose-rate, transgenerational effects, genetic effects, copy number variation (CNV), array comprehensive genomic hybridization (array CGH)

## Abstract

There is very little information on the transgenerational or genetic effects of low dose-rate ionizing radiation. We report the detection of the transgenerational effects of chronic low dose-rate irradiation in mice, at the molecular level in the whole genome, using array comparative genomic hybridization technology. We observed that the number of the mice with de novo copy number variations (specifically, deletions) was significantly increased in the offspring of C57BL/6J male mice exposed to 20 mGy/day gamma-rays for 400 days (total dose: 8000 mGy), as compared to non-irradiated controls. We did not detect any difference in the size of the de novo deletions between the irradiated and the non-irradiated groups. An analysis of the life span of the offspring suggested a possibility that de novo copy-number variations may be associated with shorter life spans.

## 1. Introduction

The transgenerational or genetic effects of ionizing radiation exposure have been a serious concern since the first scientific study, using *Drosophila* as the test system, was reported in 1927 [1]. It became more concerning after a large number of people were exposed to radiation from the atomic bombs in 1945. To date, induction of germline mutations in human populations exposed to radiation has not been clearly demonstrated [2,3,4]. The current radiation protection system [5,6] uses radiation-induced mutation rates in mice in conjunction with direct data on spontaneous human mutation rates on the assumption that, for humans, the genetic doubling dose (defined as the amount of radiation required to produce as many mutations as those occurring spontaneously in a generation) is the same as that for mice. Data on radiation-induced mutation rates in mice are derived from only a small number of studies, including those performed at Oak Ridge [7] and at Harwell [8]. These studies used classic genetic methods to analyze the induction of mutations (mostly recessive) in the loci for visible phenotypes and for enzymes in mice.

Extensive studies, including analyses of chromosome aberrations [9], blood protein polymorphisms [10], and minisatellite DNA repeats [11], have been performed on the children of the atomic bomb survivors in Hiroshima and Nagasaki, the largest cohort of the radiation-exposed population, but no significant transgenerational effects have been found [2,3,4]. A recent whole-genome sequencing (WGS) analysis of three families of atomic-bomb survivors [12] also did not find any significant change.

Offspring cohorts of cancer survivors who received radiotherapy [13], soldiers exposed to radiation from the amplifier tubes of military radar systems [14], and those exposed to radiation from the Chernobyl [15,16] and the Goiania [17,18] accidents have been the subject of transgenerational studies, wherein some cases were analyzed by using new sensitive technologies, such as array comparative genomic hybridization (CGH), single-nucleotide polymorphism (SNP) arrays, and WGS. So far, the results have been inconclusive, probably because the radiation dose or the number of individuals examined was insufficient to detect mutation events, which are induced at very low rates.

Rodent studies, usually at higher doses and, in some cases, with larger numbers of animals, have clearly demonstrated transgenerational effects of radiation exposure [2,3,4]. Recently, transgenerational effects in the offspring of male mice exposed to radiation doses of 3 or 4 Gy at high dose-rates were successfully detected by WGS and by array CGH. WGS demonstrated that radiation exposure induces clusters of single-nucleotide variants/insertions/deletions [19,20]. Array CGH showed that large (>200 kb) deletions are induced [19,21,22], as suggested in previous studies [5,23,24]. These new technologies, however, have not been applied to the analysis of transgenerational effects in the offspring of the mice chronically exposed to low dose-rate radiation. Thus, our knowledge on the transgenerational effects of low dose-rate radiation is still mainly based on the large classic mouse study performed at Oak Ridge [7].

In the present study, we analyzed the genomes of the offspring of male mice exposed chronically to low dose-rate gamma-rays through the use of array CGH, focusing on large deletions, which have been shown to be most characteristic to ionizing radiation [5,19,22,25] and to be influenced by dose-rates [24]. We detected an increase in the number of mice with de novo copy-number variations (CNVs) in the offspring of the irradiated mice.

## 2. Results

**Figure 1:**
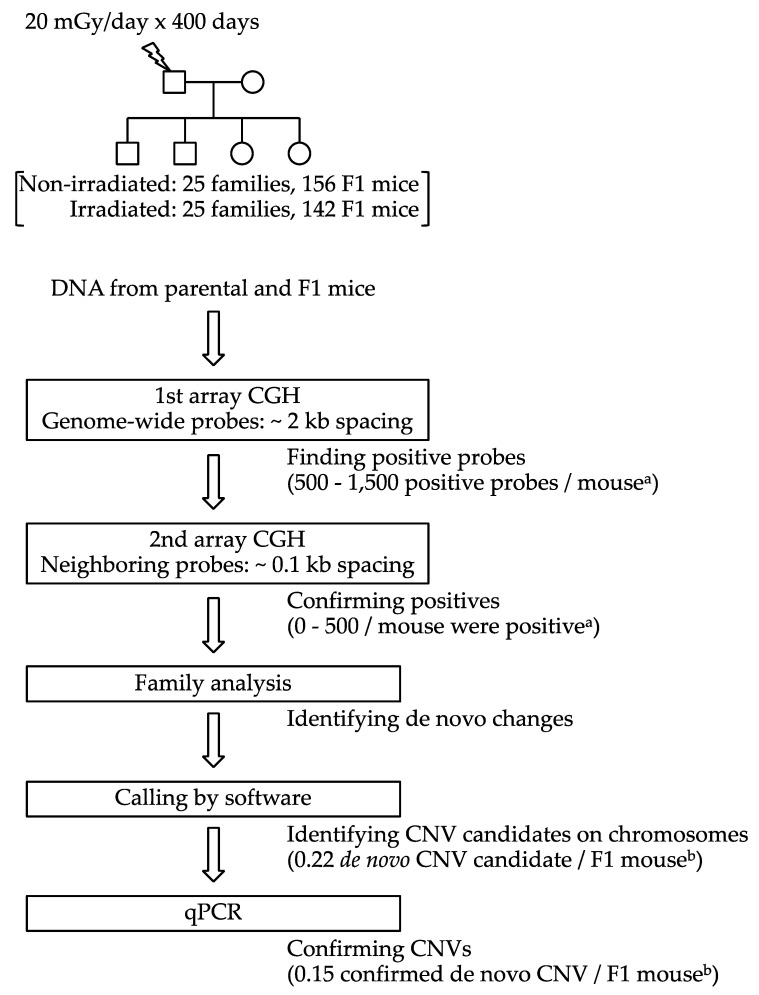
Experimental design for the identification of de novo copy number variations (CNVs). ^a^ Approximate numbers of probes for the first array CGH. ^b^ F1 mice bearing 4 or more confirmed de novo CNVs are excluded.

Figure 1 shows the schematic diagram of the experimental design. First, we analyzed the DNA samples of the parents and offspring in each family by array CGH, using ~1 million autosomal probes (probe spacing: ~2 kb). For each probe that was considered positive (with a difference in the log2 ratio between the DNA sample of the mouse and the reference DNA) in this first array CGH, we selected 10 or more neighboring probes (probe spacing: ~0.1 kb), and then we performed the second array CGH to filter out the false-positives in the first array CGH. The positive probe that passed the first array CGH screening was considered positive only if the same probe and one or more newly selected probes in the neighboring region were positive in the second array CGH screening.

To identify the positive probes that differed between parents and offspring (de novo changes), we performed family analysis, after which the locations of de novo CNV candidates in the genome were identified by using the Agilent Genomic Workbench software. Large de novo CNV candidates, with four or more positive probes in the first array CGH, were called by the software. Smaller de novo CNV candidates were identified by visual inspection of the array plots. Final confirmations of the de novo CNVs were performed by using quantitative PCR (qPCR).

**Figure 2:**
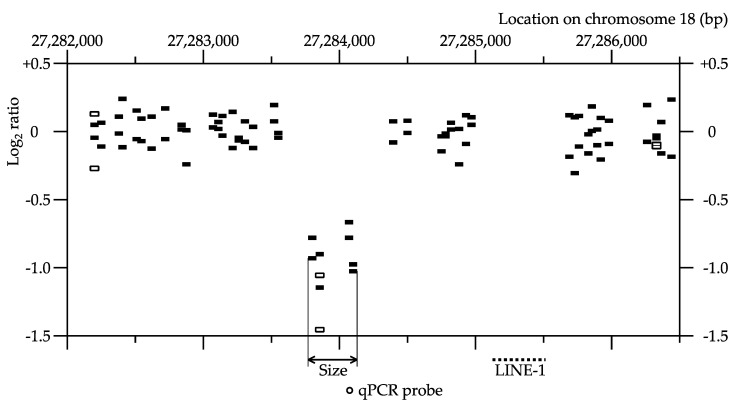
Example of a de novo CNV. The copy number profile of one of the deletions found in an F1 mouse (ID: 20mGyY1). The horizontal axis represents the chromosomal location, and the vertical axis represents the Log2 ratio of fluorescence between the sample and the reference for each probe. The results of two hybridization experiments with dye swapping are plotted separately. Open rectangles indicate the results of the first array CGH; black rectangles indicate the results of the second array CGH; the double-headed arrow indicates the estimated size of the CNV; the small open circle indicates the location of the qPCR probe (TaqMan probe); and the dotted line indicates the location of the LINE-1 repetitive sequence, which might hamper probe design.

Figure 2 is an example of a small de novo CNV, with only one positive probe in the first array CGH. In this sample deletion, the probe at position 27,283,827, but not the probes at positions 27,282,170 and 27,286,300, showed lower log2 ratios in the first array CGH. Three additional neighboring probes also showed lower log2 ratios in the second array CGH.

De novo CNVs detected by array CGH and confirmed by qPCR are listed in Table 1, including F1 mouse identity, mutation type (deletion or duplication), number of the positive probes in the first and second array CGH, estimated size, chromosomal location, and involved genes. Most of the confirmed de novo CNVs were deletions. In the non-irradiated group, 11 deletions and three duplications were found, while 31 deletions and one duplication were found in the irradiated group.

Table 2 shows the result of the classification of these confirmed CNVs and also the CNV candidates that were detected by using array CGH but were not confirmed by qPCR, according to the number of the positive probes in the first and the second array CGH. All of large CNV candidates, with two or more positive probes in the first array CGH, were confirmed by qPCR. Some of smaller CNV candidates, with only one positive probe in the first array CGH and four or less positive probes in the second array CGH, were not confirmed by qPCR. In this study, we named the CNVs found in the first array CGH with only one positive probe as “Type S” and those with two or more positive probes as “Type L”.

Table 1 and Table 2 include four mice (0mGyH5, 20mGyAA4, 20mGyN4, and 20mGyY1) with two CNVs (deletions) each. Table 3 lists five mice with four or more CNVs. We found 9–108 de novo CNV candidates (all were Type-S deletions) in each of these mice, using array CGH. Further analysis of some of these de novo CNV candidates, using qPCR, confirmed that each mouse had at least four de novo CNVs (Supplementary Materials Table S1). Assuming that the number of de novo CNVs in each mouse follows a Poison distribution, we suspect that there is a possibility that the origins of the CNVs in these five mice with multiple CNVs may be somewhat different from those in mice with only two or less CNVs.

When comparing “mutation rates” between the non-irradiated and irradiated groups, we elected to use the number of F1 mice with de novo CNV(s) (Table 4), instead of the number of de novo CNVs, in order to preclude the influence of mice with multiple CNVs. In the non-irradiated control group, 7.1% of the F1 mice (11 out of 156) had de novo deletion(s), whereas, 20.4% (29 out of 142) F1 mice from the irradiated group had de novo deletion(s). This represents a significant 2.9-fold increase in the number of F1 mice with de novo deletion(s) in the irradiated group.

CNVs detected in other studies [19,21] appear to correspond to our Type-L CNVs, so we performed similar calculations based on the number of F1 mice with Type-L CNVs. De novo Type-L deletions were found in 5.1% (8 out of 156) of the F1 mice in the non-irradiated control group and 15.5% (22 out of 142) of the F1 mice in the irradiated group, showing a significant 3.0-fold increase (Table 4).

**Figure 3:**
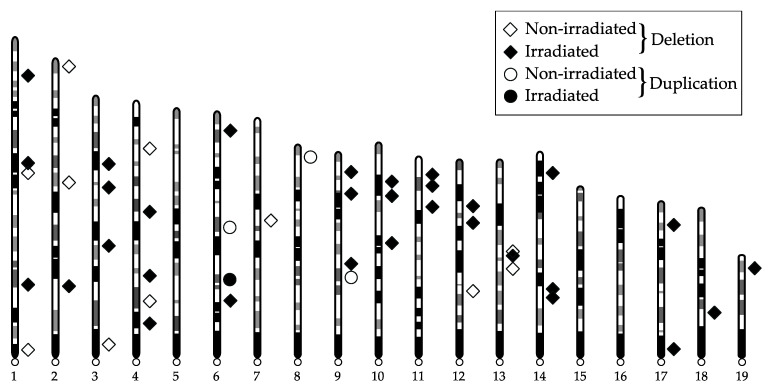
Chromosomal distribution of de novo CNVs.

The distribution of de novo CNVs on individual chromosomes is illustrated in Figure 3. We did not observe any clustering of CNVs in any specific chromosome.

**Figure 4:**
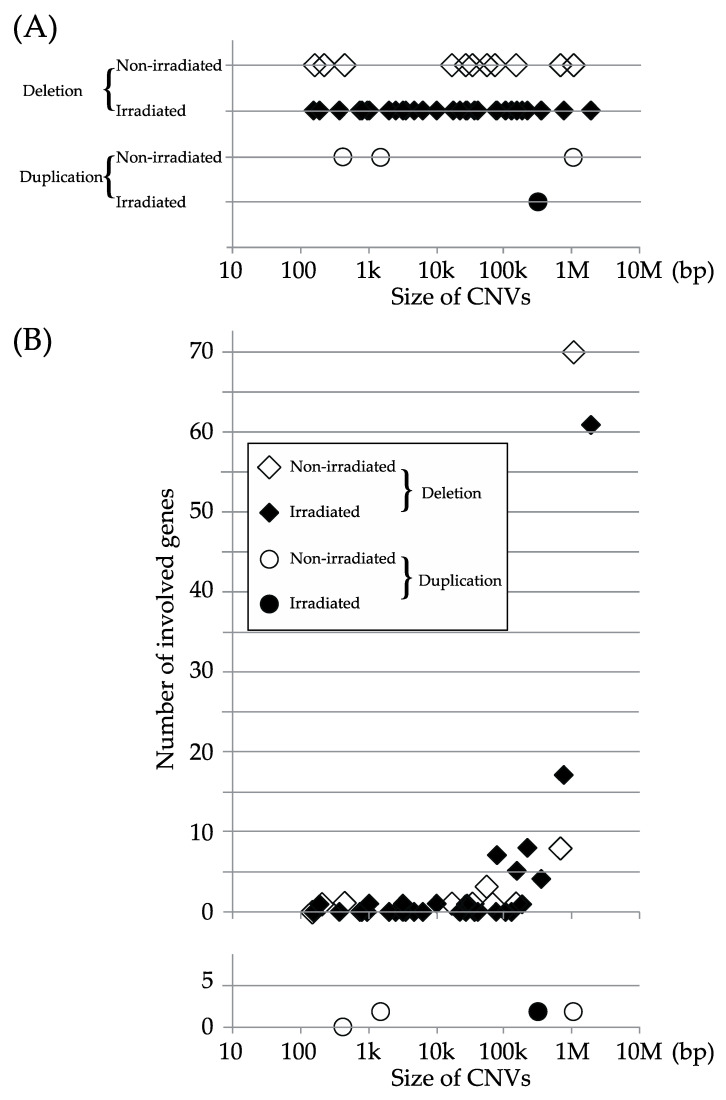
(**A**) Size of de novo CNVs. (**B**) Number of genes in the region of each de novo CNV plotted against the CNV size.

Figure 4A depicts the distribution of CNVs based on size, and Figure 4B shows the relationship between the number of genes in the region of each CNV and the size of the CNV. Most of the CNVs contained 0–2 genes, but some of large CNVs (>50,000 bp) contained 3–70 genes. The sizes of the deletions were compared between the non-irradiated and irradiated groups, using log-transformed values, since there were considerable variations in size. No significant difference was detected (*p* = 0.6758) in the size of the deletions between the groups, using the t-test (Table 5).

Since the samples used in the present study were part of a large experiment wherein both the sires (F0) and the F1 mice were allowed to live out their natural life span, we compared the life spans of the F1 mice analyzed in this study. Table 6 shows the mean life span of the F1 mice classified based on radiation exposure, sex, and the presence of CNVs. The effects of these three factors were evaluated by using the Cox proportional hazard regression analysis and are summarized in Table 7. The effect of sex on life span, expressed in terms of hazard ratio, was highly significant (*p* < 0.0001), wherein males had longer life spans than females. The effect of radiation exposure on life span was not significant (*p* = 0.1899), probably because of the small number of mice studied. The effect of CNVs on life span, however, was significant (*p* = 0.0019), suggesting a possibility that the presence of CNV(s) may be associated with shorter life spans.

## 3. Discussion

In this study, by using array CGH, we detected an increase in the number of mice bearing de novo CNVs (deletions) in the progeny of the male mice exposed chronically to low dose-rate radiation.

Recently, two groups [19,21] have reported the use of array CGH to detect increases in the number of de novo CNVs (deletions) in the progeny of the male mice exposed to acute high dose-rate radiation. Adewoye et al. [19] exposed male C57BL/6 mice to 3 Gy of X-rays at a high dose-rate and then bred them to CBA/Ca females and detected one de novo deletion (1.1%) in the progeny (*n* = 93) of the non-irradiated mice and 16 de novo deletions (9.5%) in the progeny (*n* = 169) of the irradiated male mice, using Roche NimbleGen CGH arrays (probe spacing: ~1.1 kb, Roche NimbleGen, Inc., Pleasanton, CA, USA). Asakawa et al. [21] exposed male C57BL/6J mice to 4 Gy of gamma-rays at a high dose-rate and then bred them to C3H females and detected six de novo deletions (6%) in the progeny (*n* = 100) of the non-irradiated mice and eight de novo deletions (8%) in the progeny (*n* = 100) of the irradiated mice, using Roche NimbleGen CGH arrays (probe spacing: ~1 kb or ~1.5 kb). The present study extends their findings from the effects at high dose-rates to a low dose-rate.

Our results show that the numbers of mice with Type-L deletions, which we considered similar to those detected in the previous reports, were 5.1% (8 out of 156 mice) and 15.5% (22 out of 142 mice) in the F1 mice of the non-irradiated control and the irradiated (20 mGy/day × 400 days; total dose: 8 Gy) groups, respectively. The spontaneous and induced mutation rates in these studies [19,21], including the current study, appear to be of the same magnitude, but it is difficult to conclude whether radiation exposures at acute high dose-rates are more effective in inducing deletions than chronic low dose-rates [5,6,7], because the numbers of the observed mutation events were insufficient and the dose–response relationships were not obtained in these studies. A difference has been suggested between high dose(-rate) radiation and low dose(-rate) radiation: low dose(-rate) radiation cannot fully induce the DNA damage response in cells [26], resulting in insufficient DNA repair [27]. The influence of such a phenomenon on mutation frequency, however, would be difficult to estimate, since non-homologous end joining, which is the major repair pathway of DNA double-strand breaks in higher eukaryotes and is probably responsible for the formation of many of large deletions, is error-prone or rather mutagenic [28,29].

We were unable to detect any significant difference in the size of deletions between the non-irradiated control and the low dose-rate irradiated groups. This is in sharp contrast with the results of the studies on the transgenerational effects of radiation exposures at high dose-rates, including the two studies by Adewoye et al. [19] and Kodaira et al. [22], and is in accordance with the notion that acute irradiation at high dose-rates predominantly induces larger deletions, whereas chronic irradiation at low dose-rates does not [24]. A more detailed analysis on the molecular nature of a large number of deletions is deemed necessary.

Currently, WGS techniques cannot detect deletions larger than ~50 bp in size, whereas array CGH analysis systems cannot detect deletions smaller than ~5 kb in size, resulting in a “gap” in the size of the deletions that can be identified and analyzed in mutation induction research. In an attempt to overcome this difficulty, we used arrays with very short probe spacing (~0.1 kb) in the second array CGH, so that we could detect CNVs smaller than 1 kb. Due to the prohibitive costs of the fine analysis, we had to use the arrays with larger or common probe spacing (~2 kb) for the first array CGH, such that most of small deletions would remain undetected and lost during this step. Nevertheless, the successful detection of some small deletions suggests that the use of high density probes or “tiling arrays” is one potential approach that may fill the gap in the size of detectable deletions. In the present study, we found five F1 mice bearing multiple (four or more) small (Type S) deletions. We suspect that the use of the arrays with common probe spacing in the single CGH step used in other studies might preclude the detection of such a phenomenon, which could be related to radiation-induced genome instability [30,31]. We hope that, in the future, a whole spectrum of radiation-induced mutations, from single-nucleotide changes to gross chromosomal alterations, can be revealed through the use of high density tiling arrays, together with improved WGS and chromosome analysis.

Cox proportional hazard regression analysis of the life span of the F1 mice suggested a possible association of the presence of CNV(s) with shorter life spans. Since all the analyzed F1 mice were viable, it is clear that they did not have classic dominant lethal mutations. In the list of the genes in the CNV regions (Table 1), we did not find any genes that appeared dominant deleterious. We cannot, however, discount the possibility that the alterations in copy number of some of the genes could be weakly deleterious and might slightly affect the life span. Although very preliminary, this approach might be helpful in elucidating the relationships between genotype and phenotype that have been considered to be one of the most important factors in assessing the transgenerational effects of radiation [5].

At the present time, there is no clear evidence of the transgenerational effects of radiation in humans, because of various technical difficulties. Data from laboratory animal experiments are far from sufficient. Thus, uncertainty and controversy still exist. For example, the dose–response relationships and the dose-rate effect are not certain especially in the range of low doses or low dose-rates, and there are a variety of models and opinions on this matter [30,31,32,33]. We hope that the experimental approaches described in this report will contribute towards finding a solution to these controversies.

## 4. Materials and Methods

### 4.1. Animals, Irradiation and DNA Isolation

The samples analyzed in the present study were selected from a large experiment on the transgenerational effects on life span and cancer incidence in the progeny of male mice chronically exposed to low dose-rate radiation (unpublished) referred to in a review [34]. Briefly, we irradiated specific pathogen-free male C57BL/6J/Nrs mice with ^137^Cs gamma-rays at a low dose-rate of 20 mGy/day for 400 days from 8 weeks of age. Immediately after completion of the 400-day irradiation period, the male mice were bred to non-irradiated 8-week-old virgin female C57BL/6J/Nrs mice to produce F1 mice. Tail samples were collected from dams (F0) at the time of euthanasia after weaning, and from sires (F0) and F1 mice at the time of natural death, and they were stored frozen at −80 °C until analyzed. Genomic DNA was extracted from the tail samples by a modified phenol-chloroform method with the NR-201 animal tissue DNA isolation reagent kit (Kurabo Industries Ltd., Osaka, Japan). Animal experiments were conducted according to legal regulations in Japan and the Guidelines for Animal Experiments of the Institute for Environmental Sciences (IES).

### 4.2. Array CGH

Array CGH data were deposited in the Gene Expression Omnibus (GEO) repository (accession number: GSE89425).

For the first screening by array CGH, we designed a custom 1 × 1 M array, using the eArray (currently updated to SureDesign, Agilent Technologies, Santa Clara, CA, USA) tool and database. We selected ~1 million autosomal probes (probe spacing: ~2 kb) from the ~19.1 million mouse CGH probes (probe spacing: ~0.1 kb) in the database. A small number of sex-linked probes for the determination of the sex of the mice were also selected. Array CGH experiments were performed according to the Agilent Oligonucleotide Array-Based CGH for Genomic DNA Analysis, Protocol (version 4.0.8, Agilent Technologies, Santa Clara, CA, USA). Using reference DNA from a male mouse from the in-house breeding colony at IES, we performed dye-swap hybridization experiments. An Agilent G2565BA scanner and its Feature Extraction software (version 10.7.1.1, Agilent Technologies, Santa Clara, CA, USA) and Genomic Workbench software (version 7.0, Agilent Technologies, Santa Clara, CA, USA) were used for image acquisition, data extraction, and data analysis, respectively. When both of values of |log2 ratio| for a probe, obtained in two dye-swap hybridization experiments, exceeded 0.8, the probe was considered positive (in an aberrant region). When all of the values of |log2 ratio| for two adjacent probes, in two dye-swap hybridization experiments, exceeded 0.5, these probes were also considered positive. For each of these positive probes, we selected 10 or more neighboring probes (probe spacing: ~0.1 kb) from the Agilent SureDesign database and designed custom 4 × 144 k or 8 × 60 k arrays. These arrays were used in the dye-swap hybridization experiments in the second screening. A positive probe found in the first screening was considered positive in the second screening when these two conditions were satisfied: (1) a probe positive in the first screening showed the |log2 ratio| values exceeding 0.5 in the second screening, and (2) at least one newly selected neighboring probe (within 3 probes) showed the |log2 ratio| values exceeding 0.5 in the second screening.

### 4.3. Quantitative PCR (qPCR)

We performed real-time qPCR to confirm the CNV candidates detected by array CGH, using the predesigned TaqMan Copy Number Assays probes (Thermo Fisher Scientific, Waltham, MA, USA) and the Applied Biosystems 7500 instrument (Thermo Fisher Scientific, Waltham, MA, USA). The locations of the TaqMan probes are described in Table 1. Transferrin receptor protein 1 (Tfrc1) was used as a reference gene. It is an autosomal single-copy housekeeping gene that is widely used as a reference for quantitative analysis of DNA and RNA. Amplification was performed according to the manufacturer’s protocol, in a dilution series (1.25, 2.5, and 5 ng (or 2.5, 5, and 10 ng in some experiments) of genomic DNA in a reaction volume of 20 μL), in duplex (the reference probe and the target probe were labeled with VIC [2′-chloro-7′-phenyl-1,4-dichloro-6-carboxyfluorescein] and FAM [5-(and-6)-carboxyfluorescein], respectively), and in triplicate. The relative standard curve method was used for data analyses.

### 4.4. Statistics

We compared the numbers of the mice with CNV(s) in the non-irradiated group and the irradiated group by using Fisher’s exact test. To compare the sizes of the deletions, we used geometric means instead of arithmetic means, since the sizes varied widely from 153 to 1,908,155 bp (4 orders of magnitude). The geometric means were compared by t-test on the log-transformed values after confirmation of the equality of variances by F-test. We analyzed the effects of sex, irradiation, and CNV(s) on the life span of the mice, using the Cox proportional hazard regression model. We conducted the tests on the deletion size and on the life span in R software v.4.0.0 (The R Foundation, Vienna, Austria) [35]. We used 0.05 as the threshold of statistical significance.

## Figures and Tables

**Table 1:** List of confirmed de novo CNVs.

		F1 Mouse		No. of Positive Probes		Size (bp)	Chromosomal Location (GRCm38)	qPCR Probe Location (GRCm38)	Involved Genes
		ID	Sex	1st Array CGH	2nd Array CGH				
**Non-Irradiated**	**Deletion**	0mGyX3	M	11	>50	1,103,254	Chr13:66,699,838–67,803,092	66,700,397	Gm40988, Gm48404, Gm40989, Gm7896, Gm48413, Gm40989, Vmn2r-ps108, Gm48412, Gm48414, Gm10323, Vmn2r-ps109, Cbx3-ps4, Gm5451, Gm7911, Uqcrb, Gm10767, Mterf3, Ptdss1, 4933433G19Rik, Zfp712, Gm48705, Gm46440, Zfp708, Gm28557, Gm17938, Rslcan18, Zfp759, Gm48732, Gm48733, Rsl1, Gm49646, Gm9626, Zfp455, Gm49064, Zfp458, F630042J09Rik, Zfp457, Gm48824, Zfp595, Gm28044, Zfp593, Zfp456, Gm17039, Zfp953, Gm28041, Zfp429, Gm48900, Zfp459, Zfp874a, Gm38307, Gm7928, Zfp874b, Zfp58, Gm26965, Zfp87, Zfp748, 9430065F17Rik, Zfp729b, Gm49345, Zfp729a, Zfp738, Gm48095, Gm26875, Zfp65, Gm48894, Zfp85, Gm9894, Zfp493, 4930525G20Rik
		0mGyH5 ^a^	M	7	>50	666,200	Chr2:177,329,278–177,995,478	178,041,392	Gm14414, Zfp970, Gm14403, Gm14324, Gm14322, Gm14326, Gm14327, Rps8-ps5
		0mGyH5 ^a^	M	22	>50	146,136	Chr12:40,469,827–40,615,963	40,596,701	Dock4
		0mGyG2	M	19	>50	74,519	Chr1:112,845,241–112,919,760	112,877,741	Gm8204
		0mGyS6	F	14	>50	56,272	Chr7:102,192,468–102,248,740	102,193,027	Nup98, Pgap2, Rhog
		0mGyK4	F	9	>50	34,160	Chr3:5,533,449–5,567,609	5,560,033	Pex2
		0mGyK5	F	10	>50	25,412	Chr13:54,919,455–54,944,867	54,942,291	4930526F13Rik
		0mGyJ3	F	6	>50	17,346	Chr4:128,415,985–128,433,331	128,416,544	Csmd2
		0mGyV2	M	1	2	461	Chr2:126,675,275–126,675,736	126,675,329	Gabpb1
		0mGyA3	F	1	2	212	Chr4:34,844,952–34,845,164	34,845,005	Zfp292
		0mGyO7	F	1	2	153	Chr1:7,323,388–7,323,541	7,323,321	
	**Duplication**	0mGyZ1	M	158	>50	1,100,262	Chr6:79,988,064–81,088,326	79,988,623	Lrrtm4, Gm43900
		0mGyE4	F	1	13	1535	Chr8:123,427,701–123,429,236	123,429,010	Galnt2, Def8
		0mGyG5	F	1	3	412	Chr9:49,721,288–49,721,700	49,721,288	Ncam1
**Irradiated**	**Deletion**	20mGyL5	M	575	>50	1,908,155	Chr13:63,399,812–65,307,967	65,307,023	Fancc, patch1, A930032L01 Rik, Gm30655, Gm30709, 1700024I08 Rik, Gm47387, Gm47390, Gm47417, Gm47418, Ercc6l2, Gm7695, Hsd17b3, Slc35d2, Zfp367, Gm47513, Habp4, Cdc14b, Gm46424, 1810034E14 Rik, Gm47004,Gm47003, Gm25654, Prxl2c, Zfp182, Gm47123, Gm49230, Ctsl, 1700015C15 Rik, Cdk20, Gm31218, Fam240b, Gm7712, Gm47190, Gm4810, Gm47191, Gm47193, Gm5791, Gm4935, Gm47194, Gm7065, Cntnap3, Spata31, Eif1-ps2, Prss47, Mfsd14b, Spata31d1c, Gm6888, Gm3785, Olfr465-ps1, Olfr466, Gm24130, Nlrp4f, Gm47249, Gm36445, Gm47250, Gm47251, Gm47254, Gm10775, Zfp369, Gm47258
		20mGyI5	F	200	>50	742,233	Chr10:99,214,798–99,957,031	99,959,613	Gm34777, Gm34574, Gm48884, Dusp6, Gm48089, B530045E10 Rik, Gm34921, Gad1, Gm34983, Gm35035, Gm48085, Gm20110, Gm18409, Gm35101, Gm47578, Csl, Gm47579
		20mGyL8	F	145	>50	353,159	Chr3:105,510,798–105,863,957	105,511,357	AK018929, Kcnd3
		20mGyE1	M	83	>50	235,104	Chr9:57,625,823–57,860,927	57,854,366	Csk, Cyp1a2, Cyp1a1, Edc3, Clk3, Gm17231, Arid3b
		20mGyL2	M	36	>50	145,401	Chr14:37,914,599–38,060,000	37,936,448	Gm47974
		20mGyA1	M	12	>50	133,572	Chr9:114,085,760–114,219,332	114,213,599	
		20mGyAA4 ^a^	F	34	>50	102,921	Chr1:117,376,753–117,479,674	117,377,055	
		20mGyU6	F	35	>50	76,287	Chr1:172,286,528–172,362,815	172,287,087	Igsf8, Atp1a2, Kcnj9, Gm36937, Kcnj10
		20mGyV6	F	29	>50	75,893	Chr2:45,606,923–45,682,816	45,607,482	
		20mGyW1	M	14	>50	40,175	Chr19:54,228,002–54,268,177	54,228,561	
		20mGyL4	M	8	>50	32,881	Chr6:138,709,973–138,742,854	138,741,398	
		20mGyU8	F	7	>50	29,202	Chr9:100,793,927–100,823,129	100,794,486	Stag1
		20mGyX1	M	12	>50	28,507	Chr12:92,023,170–92,051,677	92,047,867	
		20mGyV1	M	8	>50	22,299	Chr11:111,762,690–111,784,989	111,763,249	
		20mGyN4 ^a^	M	6	32	10,224	Chr6:34,932,670–34,942,894	34,941,090	Stra8
		20mGyV4	F	4	>50	9424	Chr10:107,833,661–107843085	107,834,220	Otogl
		20mGyX2	F	3	40	5965	Chr14:112,914,840–112,920,805	112,919,755	
		20mGyAA4^a^	F	2	33	4495	Chr17:81,065,337–81,069,832	81,069,694	
		20mGyU5	M	2	23	3469	Chr11:92,816,645–92,820,114	92,817,204	
		20mGyY1 ^a^	M	2	16	3370	Chr11:105,950,869–105,954,239	105,951,424	
		20mGyG2	M	2	16	3286	Chr14:40,993,918–40,997,204	40,996,166	Prxl2a
		20mGyN4 ^a^	M	1	19	2367	Chr4:50,541,636–50,544,003	50,543,175	
		20mGyG7	F	1	17	1878	Chr4:90,232,441–90,234,319	90,234,260	
		20mGyB5	F	1	3	1040	Chr10:69,420,081–69,421,121	69,420,140	Ank3-246
		20mGyG5	F	1	9	948	Chr1:44,804,456–44,805,404	44,804,512	
		20mGyX7	F	1	5	732	Chr4:21,163,257–21,163,989	21,163,816	
		20mGyH3	M	1	8	716	Chr3:119,702,208–119,702,924	119,702,263	
		20mGyY1 ^a^	M	1	4	356	Chr18:27,125,326–27,125,682	27,125,885	
		20mGyX5	F	1	2	191	Chr12:81,490,882–81,491,073	81,491,441	Synj2bp
		20mGyB2	F	1	2	156	Chr17:4,507,025–4,507,181	4,507,139	
	**Duplication**	20mGyE5	F	86	>50	305,969	Chr6:48,055,971–48,361,940	48,360,175	Zfp746, Gm16630

^a^ Mice with 2 CNVs (mice with 4 or more CNVs are not included in this table).

**Table 2:** Number of the (candidate) CNVs detected by array CGH and confirmed by qPCR.

No. of Positive Probes in 1st Array CGH		≥2 (Type L)		1 (Type S)										(Total)	
No. of Positive Probes in 2nd Array CGH				≥5		4		3		2		(Total)			
Deletion (del) or Duplication (dup)		Del	Dup	Del	Dup	Del	Dup	Del	Dup	Del	Dup	Del	Dup	Del	Dup
Non-irradiated (*n* = 156)	Detected by array CGH	8	1	0	1	1	0	0	2	13	4	14	7	22	8
	Confirmed by qPCR	8	1	0	1	0	0	0	1	3	0	3	2	11	3
Irradiated (*n* = 142)	Detected by array CGH	22	1	5	0	2	0	2	0	3	0	12	0	34	1
	Confirmed by qPCR	22	1	5	0	1	0	1	0	2	0	9	0	31	1

**Table 3:** List of the F1 mice bearing 4 or more confirmed de novo CNVs, with the number of the (candidate) CNVs detected by array CGH, analyzed by qPCR, and confirmed by qPCR.

No. of Positive Probes in 1st Array CGH			≥2 (Type L)		1 (Type S)										(Total)	
No. of Positive Probes in 2nd Array CGH					≥5		4		3		2		(Total)			
Deletion (Del) or Duplication (Dup)			Del	Dup	Del	Dup	Del	Dup	Del	Dup	Del	Dup	Del	Dup	Del	Dup
Non-irradiated	0mGyJ4 (F)	Detected by array CGH	0	0	0	0	1	0	5	0	13	0	19	0	19	0
		Analyzed by qPCR	0	0	0	0	1	0	5	0	1	0	7	0	7	0
		Confirmed by qPCR	0	0	0	0	0	0	3	0	1	0	4	0	4	0
	0mGyX5 (F)	Detected by array CGH	0	0	0	0	3	0	9	0	43	0	55	0	55	0
		Analyzed by qPCR	0	0	0	0	3	0	2	0	5	0	10	0	10	0
		Confirmed by qPCR	0	0	0	0	2	0	2	0	1	0	5	0	5	0
Irradiated	20mGyA2 (M)	Detected by array CGH	0	0	14	0	18	0	27	0	49	0	108	0	108	0
		Analyzed by qPCR	0	0	3	0	3	0	5	0	11	0	22	0	22	0
		Confirmed by qPCR	0	0	3	0	1	0	1	0	6	0	11	0	11	0
	20mGyF5 (F)	Detected by array CGH	0	0	1	0	2	0	3	0	10	0	16	0	16	0
		Analyzed by qPCR	0	0	1	0	2	0	0	0	10	0	13	0	13	0
		Confirmed by qPCR	0	0	1	0	2	0	0	0	2	0	5	0	5	0
	20mGyL1 (M)	Detected by array CGH	0	0	1	0	0	0	2	0	6	0	9	0	9	0
		Analyzed by qPCR	0	0	0	0	0	0	2	0	6	0	8	0	8	0
		Confirmed by qPCR	0	0	0	0	0	0	1	0	3	0	4	0	4	0

**Table 4:** Number of the F1 mice with confirmed de novo CNVs.

No. of Positive Probes in 1st Array CGH			≥2 (Type L)						1 (Type S)						(Total)					
Deletion (Del) or Duplication (Dup)			Del			Dup			Del			Dup			Del			Dup		
Sex		*n*	*n*	%	*p* ^a^	*n*	%	*p* ^a^	*n*	%	*p* ^a^	*n*	%	*p* ^a^	*n*	%	*p* ^a^	*n*	%	*p* ^a^
M	Non-irradiated	75	4	5.3	0.018	1	1.3	>0.1	1	1.3	>0.1	0	0.0	ND ^b^	5	6.6	0.024	1	1.3	>0.1
	Irradiated	75	13	17.3		0	0.0		3	4.0		0	0.0		14 ^c^	18.7		0	0.0	
F	Non-irradiated	81	4	4.9	>0.1	0	0.0	>0.1	2	2.5	0.085	2	2.4	>0.1	6	7.4	0.009	2	2.4	>0.1
	Irradiated	67	9	13.4		1	1.4		6	9.0		0	0.0		15	22.4		1	1.4	
Total	Non-irradiated	156	8	5.1	0.004	1	0.6	>0.1	3	1.9	0.075	2	1.2	>0.1	11	7.1	0.001	3	1.9	>0.1
	Irradiated	142	22	15.5		1	0.7		9	6.3		0	0.0		29	20.4		1	0.7	

^a^ Fisher’s exact test; irradiated group vs. corresponding non-irradiated group. ^b^ Not performed. ^c^ Two mice had both Type-L and Type-S deletions.

**Table 5:** Comparison of the sizes of the deletions found in the F1 mice of non-irradiated and irradiated groups.

	No. of Deletions	Deletion Size (bp)		
		GM ^a^	GSD ^b^	
Non-irradiated	11	18,762	12.181	*p* = 0.6758 ^c^
Irradiated	31	12,674	21.484	

^a^ Geometric mean; ^b^ geometric standard deviation; ^c^ *t*-test on log-transformed data.

**Table 6:** Life span of F1 mice ^a^.

Group of F1 Mice			No. of F1 Mice	Life Span (Days)		
				(Mean ± SD)		
M	Non-irradiated	CNV− ^b^	70	914	±	170
		CNV+ ^c^	4	859	±	161
	Irradiated	CNV-	56	824	±	163
		CNV+	16	881	±	155
F	Non-irradiated	CNV-	72	813	±	132
		CNV+	8	772	±	124
	Irradiated	CNV-	54	833	±	140
		CNV+	10	738	±	125

^a^ Very short-lived (300 days or less) mice were excluded from analysis, after they were rejected as outliers (Smirnoff–Grubbs rejection test). ^b^ Without de novo CNVs. ^c^ With de novo CNV(s).

**Table 7:** Effects of sex, irradiation, and CNVs on life span of F1 mice.

Factor	Hazard Ratio	Z Value	*p* Value
Sex ^a^	0.507	−5.40	<0.0001
Irradiation ^a^	0.851	−1.32	0.187
CNVs ^a^	0.697	−2.00	0.045

^a^ Factors of sex, irradiation, and CNVs are coded as nominal variables 0 and 1. Male, non-irradiated, and absence of de novo CNVs are assigned 0.

## Data Availability

Array CGH data were deposited in the Gene Expression Omnibus (GEO) repository (accession number: GSE89425).

## Supplementary Materials

The following is available online at https://www.mdpi.com/article/10.3390/ijms222212437/s1.
