# Supplementary material for: Increased Frequency of Copy Number Variations Revealed by Array Comparative Genomic Hybridization in the Offspring of Male Mice Exposed to Low Dose-Rate Ionizing Radiation
Source: Int J Mol Sci. 2021 Nov 18;22(22):12437. doi: 10.3390/ijms222212437 (PMC8621608; doi:10.3390/ijms222212437)
Supplement: Supplementary file 1 [file ijms-22-12437-s001.zip › ijms-1451017-supplementary.pdf]

Supplementary Materials

Table S1. List of confirmed *de novo* CNVs in the mice with 4 or more CNVs[a]

	F1 mouse		No. of positive probes		Size (bp)	Chromosomal location (GRCm38)	qPCR probe location (GRCm38)	Involved genes
	ID		1st array CGH	2nd array CGH				
Non-irradiated	0mGyJ4	F	1	2	73	Chr7:88,505,369- 88,505,442	88,505,928	
			1	3	160	Chr11:110,532,379- 11,0532,539	110,532,938	
			1	3	365	Chr12:110,004,192- 110,004,557	110,004,551	Gm34667
			1	4	450	Chr14:12,432,708- 12,433,158	12,433,267	Cadps
	0mGyX5	F	1	2	530	Chr2:80,536,082- 80,536,612	80,536,641	Nckap1
			1	4	141	Chr4:98,542,513- 98,542,654	98,543,072	Patj
			1	3	441	Chr5:150,151,600- 150,152,041	150,152,158	Fry
			1	4	272	Chr12:62,525,690- 62,525,962	62,526,249	
			1	3	286	Chr18:70,118,957- 70,119,243	70,119,516	Rab27b
Irradiated	20mGyA2	M	1	4	528	Chr2:126,675,275- 126,675,803	126,675,329	Gabpb1
			1	2	291	Chr3:116,060,017- 116,060,308	116,060,058	
			1	3	168	Chr4:54,364,673- 54,364,841	54,364,623	
			1	2	90	Chr5:66,413,774- 66,413,864	66,413,720	Apbb2
			1	2	121	Chr5:106,457,893- 106,458,014	106,457,697	Barhl2
			1	5	148	Chr6:119,015,158- 119,015,306	119,015,450	Cacna1c
			1	5	314	Chr9:33,727,971- 33,728,285	337,27,971	
			1	2	456	Chr11:45,348,967- 45,349,423	45,349,026	
			1	5	359	Chr12:88,993,149- 88,993,508	88,993,208	
			1	2	157	Chr15:48,822,820- 48,822,977	48,822,836	
			1	2	107	Chr16:8,739,256- 8,739,363	8,739,312	Usp7
	20mGyF6	F	1	2	77	Chr1:65,305,078- 65,305,155	65,304,754	Pth2r
			1	2	84	Chr2:112,492,752- 112,492,836	112,492,703	
			1	2	77	Chr3:118,562,885- 118,562,962	118,562,833	Dpyd
			1	3	153	Chr6:140,327,244- 140,327,397	140,327,079	
			1	10	1564	Chr9:80,228,807- 80,230,371	38,580,367	Myo6
	20mGyL1	M	1	2	305	Chr7:76,635,556- 76,635,861	76,635,843	Agbl1
			1	3	409	Chr9:91,348,471- 91,348,880	91,348,502	
			1	2	131	Chr13:98,143,375- 98,143,506	98,143,382	Arhgef28

			1	2	440	Chr15:62,156,950- 62,157,390	62157009	Pvt1

[a] Only deletions were found in these mice.
